# Intraoperative rapid assessment of the deep muscle surgical margin of tongue squamous cell carcinoma via Raman spectroscopy

**DOI:** 10.3389/fbioe.2024.1480279

**Published:** 2024-10-08

**Authors:** Zhongxu Li, Xiaobo Dai, Zhixin Li, Zhenxin Wu, Lili Xue, Yi Li, Bing Yan

**Affiliations:** ^1^ State Key Laboratory of Oral Diseases and National Center for Stomatology and National Clinical Research Center for Oral Diseases and Department of Head and Neck Oncology Surgery, West China Hospital of Stomatology, Sichuan University, Chengdu, Sichuan, China; ^2^ Department of Stomatology, The First Affiliated Hospital of Xiamen University, Xiamen, Fujian, China

**Keywords:** Raman spectroscopy, tongue squamous cell carcinoma, surgical margin, principal component analysis–linear discriminant analysis, diagnosis, histological grades

## Abstract

**Purpose:**

An accurate assessment of the surgical margins of tongue squamous cell carcinoma (TSCC), especially the deep muscle tissue, can help completely remove the cancer cells and thus minimize the risk of recurrence. This study aimed to develop a classification model that classifies TSCC and normal tissues in order to aid in the rapid and accurate intraoperative assessment of TSCC surgical deep muscle tissue margins.

**Materials and methods:**

The study obtained 240 Raman spectra from 60 sections (30 TSCC and 30 normal) from 15 patients diagnosed with TSCC. The classification model based on the analysis of Raman spectral data was developed, utilizing principal component analysis (PCA) and linear discriminant analysis (LDA) for the diagnosis and classification of TSCC. The leave-one-out cross-validation was employed to estimate and evaluate the prediction performance model.

**Results:**

This approach effectively classified TSCC tissue and normal muscle tissue, achieving an accuracy of exceeding 90%. The Raman analysis showed that TSCC tissues contained significantly higher levels of proteins, lipids, and nucleic acids compared to the adjacent normal tissues. In addition, we have also explored the potential of Raman spectroscopy in classifying different histological grades of TSCC.

**Conclusion:**

The PCA–LDA tissue classification model based on Raman spectroscopy exhibited good accuracy, which could aid in identifying tumor-free margins during surgical interventions and present a promising avenue for the development of rapid and accurate intraoperative techniques.

## 1 Introduction

In recent years, the incidence of oral squamous cell carcinoma has been increasing year by year, and it is considered the sixth most common global malignancy (Arathy et al., 2022; Cortina et al., 2023). Tongue squamous cell carcinoma (TSCC) is the most common oral cancer, which accounts for approximately 50% (Arathy et al., 2022; Yang et al., 2022). Although multi-disciplinary therapy has been applied in the treatment of TSCC, surgical complete resection remains the first treatment choice (Inoue et al., 2023). The primary aim of surgical treatment is the extensive resection of the tumor with no residual cancer cells, which should be achieved by removing the tumor with a margin of more than 5 mm of surrounding normal tissue (Inoue et al., 2023; Karimi et al., 2023; Spence et al., 2023). Tongue is a vital functional organ, which helps with functions such as speech, taste, and swallowing (Pai et al., 2021; Karimi et al., 2023). Surgical extensive resection would significantly impact the patients’ function and result in a decreased quality of life, although surgical reconstruction could resolve the defect of tongue tissue with free vascular flaps (Nemade et al., 2022; Cortina et al., 2023; Inoue et al., 2023). The free margin status is very important in the surgical treatment of TSCC, which should be essential for local control and minimal for functional preservation. However, it is reported that the resections of TSCC have a higher rate of positive surgical margins than other solid tumors, which shows that there is still a challenge in obtaining free margins in the resections of TSCC (Bulbul et al., 2021; Cortina et al., 2023). In the evaluation of surgical margins of TSCC, the deep muscle surgical margin has the higher rate of positive margins compared with peripheral visualized mucosal margins (Li et al., 2019; Bulbul et al., 2021). The mucosal margins can be examined and marked under direct vision from the general border of the tumor during surgery, while the deep muscle margins are not directly visible and can only be assessed by palpation and preoperative imaging (Nilsson et al., 2022). Currently, the intraoperative frozen section is considered the standard method to evaluate the surgical margins of tumors. However, there are still some challenges that persist, undermining the accuracy of the intraoperative frozen section interpretation (Li et al., 2019).

Raman spectroscopy is a vibrational spectroscopic technique that has been extensively explored for biological and biomedical applications (Falamas et al., 2021; Traynor et al., 2021). Raman spectroscopy is based on inelastic scattering that occurs in a small number of photons when the laser interacts with a sample. In the process of inelastic scattering, the scattered light caused by the molecular vibrations in a sample can be collected and displayed as the peaks in the Raman spectrum (Ibrahim et al., 2021). Raman spectroscopy can detect the changes in the biochemical compositions and the interactions of molecular structures associated with tumor tissues, which is known as the ‘molecular fingerprint’ (Talari et al., 2015; Falamas et al., 2021). In previous studies, Raman spectroscopy combined with different algorithms has been successfully used to detect oral premalignant or cancerous tissues with high accuracies (Sharma et al., 2021a; Chang et al., 2023). Compared with other vibrational spectroscopies, Raman spectroscopy is a rapid, label-free, real-time, and non-invasive method for tissues and liquid detection with the high resolution, low water interference, and uncomplicated sample preparation, (Falamas et al., 2021; Traynor et al., 2021), which is suitable for the intraoperative detection of the surgical deep margin of TSCC.

Discrimination between cancerous and normal muscle tissues plays a key role in the evaluation of the deep surgical margins. In the present study, we aim to apply Raman spectroscopy to discriminate cancerous and muscle tissues in the deep surgical margin regions of TSCC, in order to establish a rapid and intraoperative method to detect and evaluate the deep surgical margins of TSCC.

## 2 Materials and methods

### 2.1 Patients

A total of 15 patients with TSCC participated in this study. Patients were diagnosed by two experienced pathologists according to the TSCC diagnostic criterion. All patients did not receive any treatment prior to this study and did not have any systemic diseases or a history of drug abuse. The patients were informed, and their consents were obtained prior to the start of this study. This study was approved by the Institution Review Board of Hospital, and the Declaration of Helsinki protocols were followed during this study. The clinical information on patients is recorded in Table 1.

**TABLE 1:** Clinical information on patients.

No.	Age (year)	Gender	Histological grade	TNM classification
1	67	Male	MDSCC	T2N1M0
2	67	Male	MDSCC	T2N1M0
3	68	Male	MDSCC	T2N1M0
4	67	Male	MDSCC	T2N1M0
5	68	Male	MDSCC	T2N1M0
6	66	Female	WDSCC	T2N0M0
7	71	Male	PDSCC	T2N0M0
8	75	Male	MDSCC	T3N0M0
9	68	Male	PDSCC	T4N2M0
10	45	Male	MDSCC	T4N2M0
11	70	Female	WDSCC	T1N2M0
12	64	Female	PDSCC	T1N2M0
13	75	Male	WDSCC	T2N0M0
14	29	Male	WDSCC	T1N0M0
15	55	Female	WDSCC	T2N0M0

### 2.2 Tissue samples

The cancerous tissues and normal muscle tissues were obtained from the patients undergoing surgical treatment at the First Affiliated Hospital of Xiamen University. The cancerous tissues were obtained from the tumor of TSCC patients, and the normal muscle tissues were obtained from the 5-mm muscle tissue regions beyond the tumor border. Then, the cancerous and normal muscle tissues were divided into two parts. One part of the samples was produced into the routine hematoxylin–eosin-stained sections for pathological verification and diagnosis. The other part of the samples was cut into parallel 8-um-thick sections using a freezing microtome. The 8-um-thick sections were mounted on the custom pure aluminum chips for Raman spectroscopic investigation. There were a total of 60 sections for Raman spectroscopic examination made from 15 TSCC patients, including 30 sections of cancerous tissues and 30 sections of normal muscle tissues.

### 2.3 Raman spectroscopy

Raman spectra of cancerous tissues and normal muscle tissues were carried out using a Nanophoton Raman-11 laser Raman microscope (Nanophoton, Japan) using a 532-nm excitation laser, ×20 objective, and a liquid nitrogen-cooled CCD. The excitation laser beam was focused on the sample through the X20 0.45 NA Nikon lens. Samples were placed in the motorized XYZ stage to ensure accurate positioning, and the Raman spectra of the four interested regions around the center of the sample were recorded with a spectral resolution of 1 cm^−1^.

### 2.4 Data analysis

Before the multivariate analysis of the Raman spectral data, the raw Raman spectra were processed to remove autofluorescence backgrounds, the noisy interferences, and oversaturated spectra and were smoothed by using the Vancouver Raman algorithm (Zhao et al., 2007). Then, the baseline correction and normalization of the Raman spectra were carried out by using OriginPro 8.0 software (OriginLab, Northampton, MA, United States) before the multivariate analysis. In addition, the mean spectra of cancerous tissues and normal muscle tissues and the subtracted Raman of different tissues were obtained through OriginPro 8.0 software.

In the multivariate analysis process, principal component analysis (PCA) was applied to reduce the dimensions of numerous Raman data and generate principal components. Then, linear discriminant analysis (LDA) was employed to classify different Raman spectra of cancerous tissues and normal muscle tissues. In this study, the generated principal components that account for more than 90% of the total variables in Raman spectra were selected as statistical variables in LDA. The leave-one-out cross-validation was employed in order to estimate and evaluate the prediction performance of the classification model established by PCA and LDA. In this study, the classification model was not only employed to discriminate Raman spectra of cancerous tissues and normal muscle tissues but also applied to classify the Raman spectra of TSCC tissues with different degrees of differentiation.

## 3 Results

The testing process was obtaining tissue sections from TSCC and normal muscle tissues and performing pathological diagnosis and Raman spectral analysis. A total of 240 Raman spectra were obtained successfully from the samples using the Raman microscope. These spectra were grouped according to the histological grades for the next analysis.

### 3.1 Raman spectral difference and discrimination of TSCC and normal muscle tissues

**FIGURE 1:**
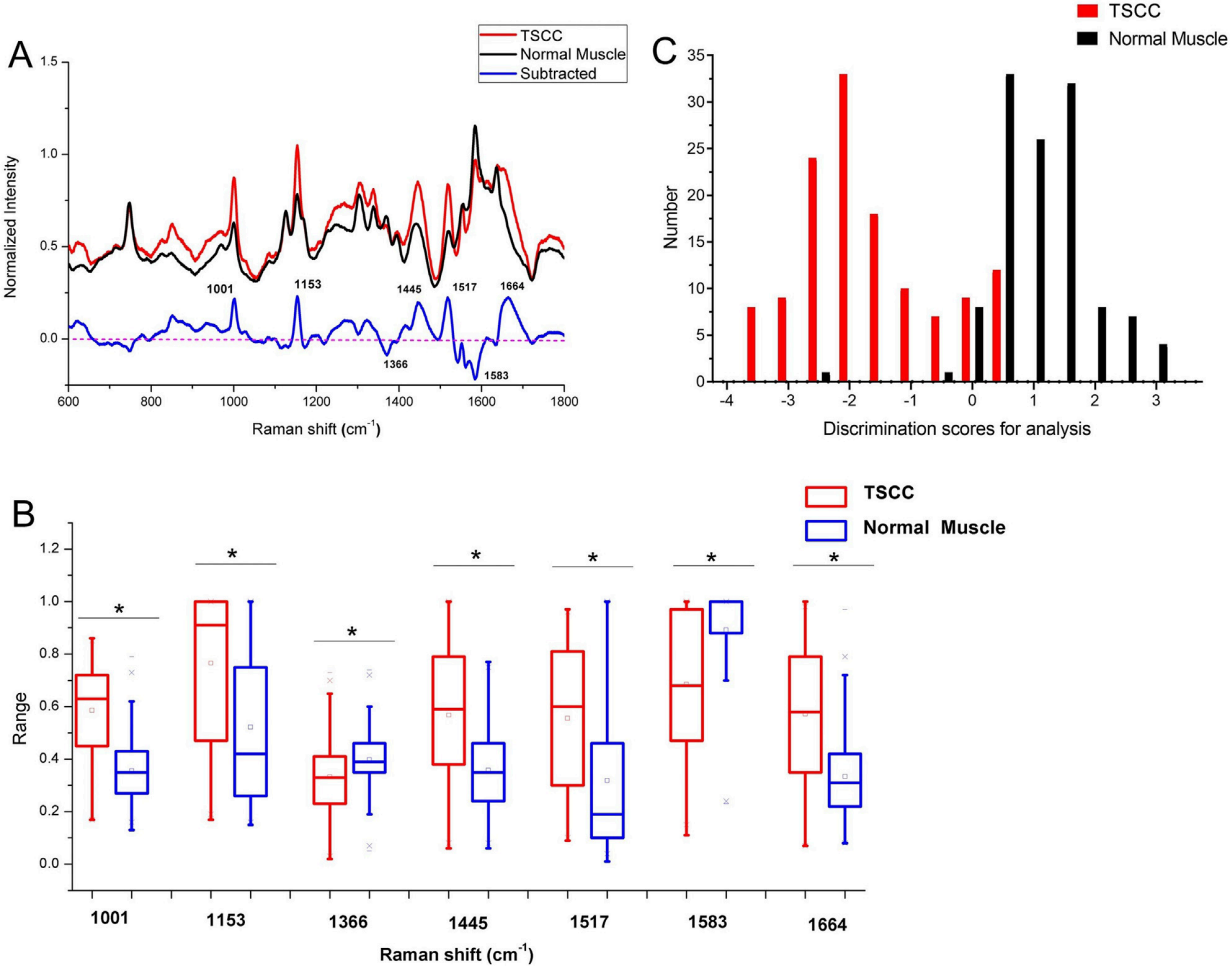
**(A)** Subtracted Raman spectrum of TSCC and normal muscle. **(B)** Independent-sample t-test’s results of the subtracted spectra peaks of different spectra. Asterisk (*) indicates significant difference (*p* < 0.05). **(C)** Histogram of discrimination scores of TSCC and normal muscle. Abbreviations: TSCC, tongue squamous cell carcinoma.

According to the pathological diagnosis, a total of 240 Raman spectra were classified into the normal muscle and TSCC groups. There were 120 spectra in the normal muscle group and 120 spectra in the TSCC group. Comparing the mean spectra of different tissues, the main Raman peaks appeared at similar wavelengths. However, marked differences between the mean spectra were observed, such as the Raman shifts, intensities, and height and width of peaks (Figure 1A). The subtracted spectra showed that there were significant enhancements in the peaks at Raman shifts of 1,001, 1,153, 1,445, 1,517, and 1,664 cm^−1^. The characteristic assignments of different peaks of TSCC and normal muscle tissues are shown in Table 2 (Zúñiga et al., 2019; Buchan et al., 2021; David et al., 2022; Depciuch et al., 2023). According to the reported literature reports and previous studies, these peaks were assigned to C–C phenylalanine ring breathing modes, carotenoids, CH_2_, CH_3_ bending in proteins and lipids, C–N stretching modes, and nucleic acid modes. Meanwhile, the subtracted spectra also showed some significant decreases in the peaks at Raman shifts of 1,366 and 1,583 cm^−1^ of the mean spectra of the TSCC tissues. These peaks were assigned to adenine, guanine, and thymine in DNA and amide II. Compared with the normal muscle tissues, the major increased peaks in the TSCC tissues were assigned to the molecular structures of the nucleic acids and proteins. Similarly, we have analyzed the mean Raman spectra of TSCC and normal muscle tissue of each patient. The mean spectra and subtracted spectra are presented in Supplementary Figure S1 and Supplementary Figure S2. Most of the subtracted spectra of the 15 patients showed some significant increases in the peaks at Raman shifts of 851, 1,001, 1,153, 1,445, 1,517, 1,656, and 1664 cm^−1^, which also showed some decreases in the peaks at Raman shifts of 746, 1,366 and 1583 cm^−1^. The above results were similar to the mean Raman spectra of all patients. However, the subtracted spectra of patient nos 6, 12, and 14 differed somewhat from the other spectra between peaks 1,400 and 1,700 cm^−1^.

**TABLE 2:** Assignments of Raman peaks of TSCC and normal muscle tissues.

Peak (cm^−1^)	Assignment
746	Ring breathing mode of DNA and RNA bases and symmetric breathing of tryptophan (protein assignment)
851	C–C stretching mode from multiple sites in collagen backbone, α-helix, valine, and proline
1,001	C–C phenylalanine ring breathing mode
1,123	C–N stretching mode of proteins
1,147 and 1,153	Carotenoids
1,318	CH deformation in proteins
1,357, 1,366 and 1,370	Adenine, guanine, and thymine in DNA
1,445 and 1,449	CH_2_ and CH_3_ bending in proteins and lipids
1,517, 1,519 and 1,542	C–N stretching mode
1,581, 1,583 and 1,587	Amide II
1,612	Amide I
1,656	C = C of lipids
1,664 and 1,668	Nucleic acid modes, indicator of tissue DNA content, and amide I

Figure 1B shows the results of an independent-sample *t*-test comparing the Raman spectra peaks between TSCC tissues and normal muscle tissues at specific Raman shift values. Each pair of boxplots (red for TSCC and blue for normal muscle) represents the range of Raman spectra intensity at specific Raman shift values labeled along the *x*-axis (1,001, 1,153, 1,366, 1,445, 1,517, 1,583, and 1,664 cm^−1^). The results show that the difference in Raman intensity between TSCC and normal muscle tissue observed at these Raman shifts is statistically significant (indicated by an asterisk (*) above the box plot). In other words, the molecular environment of TSCC tissue is significantly different from normal muscle tissue.

As the result of the PCA-LDA process, 99 of 120 spectra in the TSCC group and 118 of 120 spectra in the normal muscle group were classified into the accurate groups, respectively. The accuracy of the normal muscle group classification that reached 98.3% was the highest, and the total accuracy of the diagnostic model established by PCA–LDA was 90.4%. The total accuracy of the leave-one-out cross-validation was 89.2%, which demonstrated the stable prediction performance of the diagnostic mode (Table 3). The histogram of discrimination scores of the two groups demonstrated a clear classification (Figure 1C).

**TABLE 3:** Results of the classification of TSCC and normal muscle stage groups by PCA–LDA.

Method	Class	Predicted group		Total
		TSCC	Normal muscle	
PCA–LDA count (%)	TSCC	99 (82.5%)	21 (17.5%)	120 (100%)
	Normal muscle	2 (1.7%)	118 (98.3%)	120 (100%)
Cross-validated count (%)	TSCC	97 (80.8%)	23 (19.2%)	120 (100%)
	Normal muscle	3 (2.5%)	117 (97.5%)	120 (100%)

### 3.2 Raman spectral difference and discrimination of different histological grades of TSCC and normal muscle tissues

**FIGURE 2:**
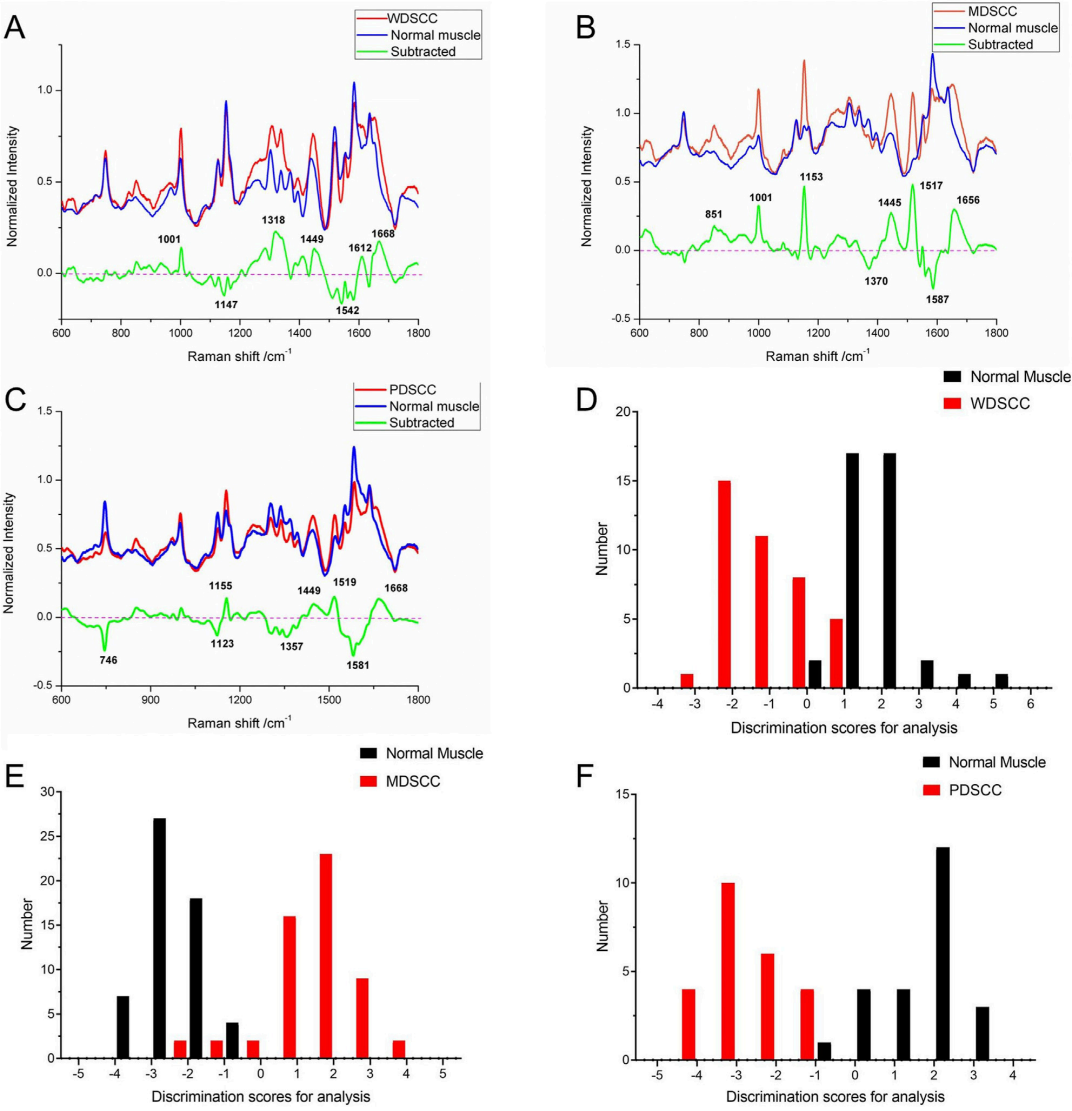
**(A)** Subtracted Raman spectrum of WDSCC and normal muscle. **(B)** Subtracted Raman spectrum of MDSCC and normal muscle. **(C)** Subtracted Raman spectrum of PDSCC and normal muscle. **(D)** Histogram of discrimination scores of WDSCC and normal muscle. **(E)** Histogram of discrimination scores of MDSCC and normal muscle. **(F)** Histogram of discrimination scores of PDSCC and normal muscle. Abbreviations: WDSCC, well-differentiated squamous cell carcinoma; MDSCC, moderately differentiated squamous cell carcinoma; PDSCC, poorly differentiated squamous cell carcinoma.

According to the histological grades, a total of 240 Raman spectra were classified into three groups, including 40 spectra of well-differentiated squamous cell carcinoma (WDSCC), 56 spectra of moderately differentiated squamous cell carcinoma (MDSCC), 24 spectra of poorly differentiated squamous cell carcinoma (PDSCC), and 120 spectra of normal muscle tissues corresponding to each TSCC group. The characteristic assignments of different peaks of different histological grades of TSCC are shown in Table 4 (Yan et al., 2011; Yan et al., 2015; Fousková et al., 2023). Compared with different mean spectra, the intensities of peaks at 1,001, 1,318, 1,449, 1,612, and 1,668 cm^−1^ in the Raman spectra increased from the WDSCC group to the normal muscle group (Figure 2A). These peaks were assigned to the C–C phenylalanine ring breathing mode, CH deformation in proteins, CH_2_, CH_3_ bending in proteins and lipids, and nucleic acid modes. Hstretching mode from multiple sites inowever, there were also decreased peaks at 1,147 and 1,542 cm^−1^ in the spectra, which were assigned to the carotenoids and C–N stretching mode. These differences in peaks suggested the changes in the biochemical components such as nucleic acid, proteins, and lipids.

**TABLE 4:** Assignments of Raman peaks of WDSCC, MDSCC, and PDSCC.

Peak (cm^−1^)	Assignment
746 and 748	Ring breathing mode of DNA and RNA bases and symmetric breathing of tryptophan (protein assignment)
1,001 and 1,003	C–C phenylalanine ring breathing mode
1,152 and 1,153	Carotenoids
1,314	CH deformation in proteins
1,445	CH_2_ and CH_3_ bending in proteins and lipids
1,517 and 1,518	C–N stretching mode
1,587 and 1,608	Amide II
1,651 and 1,653	C = C of lipids

Meanwhile, the subtracted spectrum of the MDSCC and normal muscle groups is shown in Figure 2B. It showed some significant increases in the peaks at Raman shifts of 851, 1,001, 1,153, 1,445, 1,517, and 1,656 cm^−1^ of the mean spectrum. These peaks were assigned to the C–C stretching mode from multiple sites in the collagen backbone, α-helix, valine, proline, C–C phenylalanine ring breathing mode, carotenoids, CH_2_, CH_3_ bending in proteins and lipids, C–N stretching modes, and C = C of lipids. Meanwhile, the subtracted spectrum also showed some decreases in the peaks at Raman shifts of 1,370 and 1,587 cm^−1^, which were assigned to adenine, guanine, and thymine in DNA and amide II. These differences of peaks suggested that the content of nucleic acid and protein and the amide II components were changed.

When comparing PDSCC and normal muscle groups in the subtracted spectrum (Figure 2C), there are not only positive peaks but also negative peaks. The positive peaks at Raman shifts of 1,155, 1,449, 1,519, and 1,668 cm^−1^ can be assigned to carotenoids, CH_2_, CH_3_ bending in proteins and lipid C–N stretching mode, and nucleic acid modes, respectively. These positive peaks suggest that the contents of proteins, lipids, and nucleic acids in the PDSCC were higher than that in normal muscle tissues. The negative peaks suggest that the contents of nucleic acid and amide II components may decrease at 746, 1,123, 1,357, and 1581 cm^−1^, which are assigned to the ring breathing mode of DNA and RNA bases, symmetric breathing of tryptophan, and C–N stretching modes of proteins, adenine, guanine, and thymine in DNA and amide II.

The classification and diagnosis of the WDSCC and normal muscle group were carried out using the PCA–LDA method. The results showed that 35 of 40 spectra in the WDSCC group and 38 of 40 spectra in the normal muscle group were classified into the accurate groups, respectively. The accuracy of WDSCC group classification reached 87.5%, and the accuracy of normal muscle group classification reached 95.0%. The total accuracy of the diagnostic model established by PCA-LDA was 91.3%. The total accuracy of the leave-one-out cross-validation was 85.0%, which demonstrated the stable prediction performance of the diagnostic model (Table 5). The histogram of discrimination scores demonstrated a clear separation of the spectra of the WDSCC and normal muscle groups (Figure 2D).

**TABLE 5:** Results of the classification of WDSCC and normal muscle stage groups by PCA–LDA.

Method	Class	Predicted group		Total
		WDSCC	Normal muscle	
PCA–LDA count (%)	WDSCC	35 (87.5%)	5 (12.5%)	40 (100%)
	Normal muscle	2 (5.0%)	38 (95.0%)	40 (100%)
Cross-validated count (%)	WDSCC	31 (77.5%)	9 (22.5%)	40 (100%)
	Normal muscle	3 (7.5%)	37 (92.5%)	40 (100%)

The classification and diagnosis of the MDSCC and normal muscle groups were also carried out using the PCA–LDA method. As a result of the PCA–LDA process, 52 of the 56 spectra were in the MDSCC group and 56 spectra were in the normal muscle group. The accuracy of MDSCC group classification reached 92.9%, and the accuracy of normal muscle group classification reached 100.0%. The total accuracy of the diagnostic model established by PCA–LDA was 96.4%. The total accuracy of the leave-one-out cross-validation was 93.8%, which demonstrated the stable prediction performance of the diagnostic model (Table 6). The histogram of discrimination scores demonstrated a clear separation of the spectra of the MDSCC and normal muscle groups (Figure 2E).

**TABLE 6:** Results of the classification of MDSCC and normal muscle stage groups by PCA–LDA.

Method	Class	Predicted group		Total
		MDSCC	Normal muscle	
PCA–LDA count (%)	MDSCC	52 (92.9%)	4 (7.1%)	56 (100%)
	Normal muscle	0 (0.0%)	56 (100.0%)	56 (100%)
Cross-validated count (%)	MDSCC	51 (91.1%)	5 (8.9%)	56 (100%)
	Normal muscle	2 (3.6%)	54 (96.4%)	56 (100%)

The classification and diagnosis of the PDSCC and normal muscle group were also carried out using the PCA–LDA method. As a result of the PCA–LDA process, 24 of the 24 spectra were in the PDSCC group and 23 spectra were in the normal muscle group. The accuracy of PDSCC group classification reached 100.0%, and the accuracy of normal muscle group classification reached 95.8%. The total accuracy of the diagnostic model established by PCA–LDA was 97.9%. The total accuracy of the leave-one-out cross-validation was 91.7%, which demonstrated the stable prediction performance of the diagnostic model (Table 7). The histogram of discrimination scores demonstrated a clear separation of the spectra of the PDSCC and normal muscle groups (Figure 2F).

**TABLE 7:** Results of the classification of PDSCC and normal muscle stage groups by PCA–LDA.

Method	Class	Predicted group		Total
		PDSCC	Normal muscle	
PCA–LDA count (%)	PDSCC	24 (100.0%)	0 (0.0%)	24 (100%)
	Normal muscle	1 (4.2%)	23 (95.8%)	24 (100%)
Cross-validated count (%)	PDSCC	22 (91.7%)	2 (8.3%)	24 (100%)
	Normal muscle	2 (8.3%)	22 (91.7%)	24 (100%)

### 3.3 Raman spectral difference and discrimination of WDSCC, MDSCC, and PDSCC

**FIGURE 3:**
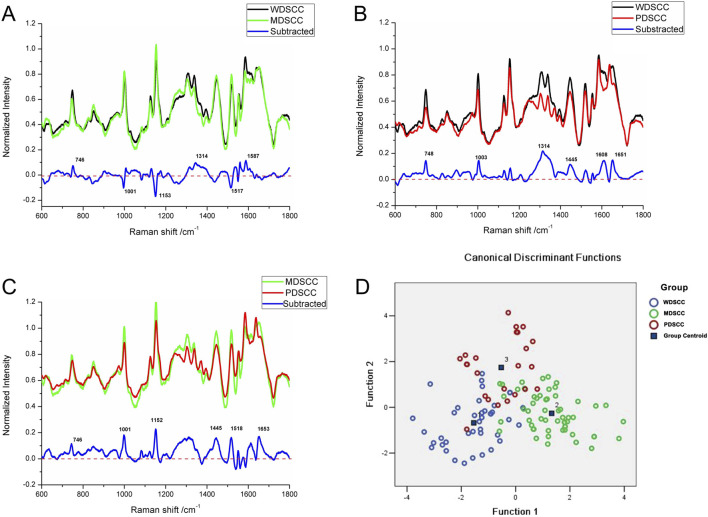
**(A)** Subtracted Raman spectrum of WDSCC and MDSCC. **(B)** Subtracted Raman spectrum of WDSCC and PDSCC. **(C)** Subtracted Raman spectrum of MDSCC and PDSCC. **(D)** Two-dimensional scatter plot diagram of the WDSCC, MDSCC, and PDSCC groups. Abbreviations: WDSCC, well-differentiated squamous cell carcinoma; MDSCC, moderately differentiated squamous cell carcinoma; PDSCC, poorly differentiated squamous cell carcinoma.

Compared with the WDSCC and MDSCC groups in the subtracted spectrum (Figure 3A), the main Raman peaks, which showed some significant increases, appeared at 746, 1,314, and 1,587 cm^−1^ from the 600 cm^−1^ to 1800 cm^−1^ Raman shift region. These peaks were assigned to the ring breathing mode of DNA and RNA bases, symmetric breathing of tryptophan (protein assignment), and CH deformation in proteins and amide II. Meanwhile, the subtracted spectrum also showed some decreases in the peaks at Raman shifts of 1,001, 1,153 and 1,517 cm^−1^, which were assigned to the C–C phenylalanine ring breathing mode, carotenoids, and C–N stretching mode. These differences in peaks suggested that there were more nucleic acid, protein, and amide I components but less lipids in the WDSCC tissues than those in the MDSCC tissues. When comparing WDSCC and PDSCC groups in the subtracted spectrum (Figure 3B), the intensities of peaks at 748, 1,003, 1,314, 1,445, 1,608, and 1,651 cm^−1^ in the Raman spectra increased. These peaks were assigned to the ring breathing mode of DNA and RNA bases, symmetric breathing of tryptophan (protein assignment), C–C phenylalanine ring breathing mode, CH deformation in proteins, CH_2_, CH_3_ bending in proteins and lipids, amide II, and C = C of lipids. These differences in peaks suggested that there were more nucleic acid, protein, lipids, and amide II components in the WDSCC tissues than those in the PDSCC tissues. Comparing MDSCC and PDSCC groups in the subtracted spectrum (Figure 3C), some significant increases were observed in the peaks at Raman shift 746, 1,001, 1,152, 1,445, 1,518, and 1,653 cm^−1^ of the mean spectrum. These peaks were assigned to the ring breathing mode of DNA and RNA bases, symmetric breathing of tryptophan (protein assignment), C–C phenylalanine ring breathing mode, carotenoids, CH_2_, CH_3_ bending in proteins and lipids, C-N stretching mode, and C = C of lipids. These differences in peaks suggested that there were more nucleic acid, protein, and lipids in the MDSCC tissues than those in the PDSCC tissues.

To classify and discriminate the 120 spectra of different histological grade groups, the PCA–LDA algorithm was employed. As the result of the PCA–LDA process, 33 of the 40 spectra in the WDSCC group, 48 of the 56 spectra in the MDSCC group, and 19 of the 24 spectra in the PDSCC group were classified into the corresponding groups. The accuracy of MDSCC group classification that reached 85.7% was the highest, and the total accuracy of the diagnostic model established by PCA–LDA was 83.3%. The total accuracy of the leave-one-out cross-validation was 77.5%, which demonstrated the stable prediction performance of the diagnostic model (Table 8). The two-dimensional scatter plot diagram of discrimination scores demonstrated a clear classification of the three groups (Figure 3D).

**TABLE 8:** Results of the classification of WDSCC, MDSCC, and PDSCC stage groups by PCA–LDA.

Method	Class	Predicted group			Total
		WDSCC	MDSCC	PDSCC	
PCA–LDA count (%)	WDSCC	33 (82.5%)	2 (5.0%)	5 (12.5%)	40 (100%)
	MDSCC	2 (3.6%)	48 (85.7%)	6 (10.7%)	56 (100%)
	PDSCC	4 (16.7%)	1 (4.2%)	19 (79.2%)	24 (100%)
Cross-validated count (%)	WDSCC	32 (80.0%)	3 (7.5%)	5 (12.5%)	40 (100%)
	MDSCC	2 (3.6%)	45 (80.4%)	9 (16.1%)	56 (100%)
	PDSCC	5 (20.8%)	3 (12.5%)	16 (66.7%)	24 (100%)

## 4 Discussion

To date, TSCC accounts for over 50% of oral cavity cancers, with patients typically presenting with locally advanced stages of the disease. The primary treatment modality is comprehensive surgical excision, necessitating adequately expanded surgical margins to eliminate any residual tumor cells. However, the tongue performs critical physiological roles, significantly influencing patients' quality of life (Watanabe et al., 2023). Consequently, precise delineation of surgical margins for tongue cancer is imperative. Current practices for determining tumor margins rely heavily on visual inspection and palpation, which usually lead to insufficient histopathological margins (Aaboubout et al., 2021). In particular, the deep surgical margins are the most difficult place to achieve clear margins due to the inability to get direct vision. Intraoperative frozen section analysis serves as a crucial tool for the histopathological examination of suspect regions during surgery, aiding in the assessment of the mucosal margins’ extent. The efficacy of this diagnostic approach is contingent upon the pathologist’s expertise, and it is very time-consuming and laborious. In addition, comparing to the more readily assessable peripheral mucosal margins, the deep muscle surgical margin exhibits a higher rate of positive diagnoses, underscoring its critical importance in the evaluation of TSCC surgical margins (Bulbul et al., 2021). Thus, an intraoperative tool that can adjunct an intraoperative frozen section for diagnosis that provides a real-time and objective evaluation of the margins (especially in the deeper muscle tissue margins) of more than 5 mm of the surrounding normal tissue may improve the prognosis of patients with TSCC.

Raman spectroscopy, a nondestructive vibrational spectroscopy technique, leverages alterations in biochemical components for disease identification and diagnosis without the necessity for tissue pretreatment or labeling (Zhang et al., 2022). Raman spectroscopy combined with different algorithms has been successfully used to detect parotid neoplasms or cancerous tissues with high accuracies in our previous studies (Xue et al., 2014; Yan et al., 2015; Xue et al., 2018). Raman spectroscopy can detect the differences and changes in the biochemical components and histopathological structures in the molecular level, which is described as “molecular fingerprint.” The chemical composition, molecular structures, and expressions of some proteins always change during different histological grades in the TSCC tissue (Cals et al., 2016). In this study, tissue sections were obtained from cancerous and normal muscle tissues and classified by performing pathological diagnosis and then detected by Raman spectral analysis. A total of 240 Raman spectra were obtained successfully from the samples using the Raman microscope. These Raman spectra were used to demonstrate differences in “molecular fingerprints” between different tissues. There were many algorithms for analyzing and classifying spectral data (Sharma et al., 2023; Fuentes et al., 2024). SVM was classified in high-dimensional space, which was effective but had high computational complexity. Neural networks were not superior in dealing with linear problems and required large amounts of training data and computational resources. Comparing with these algorithms, PCA–LDA had been extensively applied for the accurate identification of oral cancerous tissues and had the most applications in Raman spectral classification with better performance (Xue et al., 2018). In our study, PCA was applied to reduce the dimensionality of Raman spectra by transforming the data into a set of orthogonal principal components that capture the most variance in the data. After PCA reduced the dimensionality, LDA was applied to find the linear combinations of features that best separate different classes. This combination enhanced the ability to distinguish between classes with high accuracy. It was the first time that PCA–LDA can successfully discriminate and classify not only the spectra of TSCC and normal muscle tissues but also the spectra of TSCC tissues with different degrees of differentiation. In the process of cross-validation, the leave-one-out method was employed to test the results. The cross-validated results were similar to the results of the classification model, which suggested that the PCA–LDA algorithm was suitable and reliable to analyze and classify the Raman spectral data.

In our study, the mean spectrum of TSCC exhibits higher peaks compared to normal muscle tissue. We found that the peak at 1,001 cm^−1^ can be assigned to the characteristic phenylalanine of the protein. The intensity at peak 1,153 cm^−1^ was higher for both types of tissues, but TSCC tissues exhibited more intense carotenoid peaks. Rau et al. reported that the cancer intensity was significantly higher at this peak in the Raman mode than in healthy tissues, which is in good agreement with our results (Rau et al., 2017). The cancerous spectral features included a broad and distinct amide I peak at 1,664 cm^−1^, a CH_2_ bending peak at 1,445 cm^−1^, a C–N peak at 1,517 cm^−1^, and a distinct phenylalanine peak at 1,001 cm^−1^, all of which were significantly associated with the increased protein content in TSCC tissues. The literature confirms that the peaks in malignant tissues are mainly driven by proteins, which may be related to the value added, metabolic changes, and extracellular matrix changes in malignant cells (Sahu et al., 2016; Sharma et al., 2021b). In addition, TSCC tissues showed higher intensity at 1,664 cm^−1^ (nucleic acid modes), indicating active nucleic acid metabolism and high nucleic acid content in TSCC patients. This is consistent with previous reports (Sahu et al., 2015). However, the intensity was lower at 1,366 cm^−1^ (adenine, guanine, and thymine in DNA). It is considered that there may be changes in the chromatin structure in cancer tissues, and the compactness of chromatin may affect the vibrational properties of adenine, guanine, and thymine in DNA, making their Raman intensity weaker.

The substantial differences between cancerous and healthy tissues led to a high discrimination rate in this study. The total accuracy of the diagnostic model established by PCA–LDA reached 90.4%. When comparing Raman spectral difference and discrimination between the histological grade group and normal muscle tissue group and among different histological grade groups, the lowest accuracy of the diagnostic model reached 91.3% and 93.3%, respectively. The cross-validated results were similar to the results of the classification model, which suggested that the PCA–LDA algorithm was suitable and reliable to analyze and classify the Raman spectral data on TSCC. We conclude that the highest accuracy of the PCA–LDA classification model would be helpful in achieving adequate resection margins and that such clinical implementation is technically feasible.

We analyzed the mean Raman spectra of TSCC and normal muscle tissue of each patient and found that their characteristic peaks were similar to those of the mean spectra of all patients, especially when compared with the mean spectra of the same histological grading. However, the magnitude of the subtracted spectra varied between these patients, such as patient nos 6, 12, and especially 14. We believe that the most important reasons for this difference are the degrees of differentiation of the tissues and individual differences. The composition and structure of molecules in TSCC tissues with different degrees of differentiation are different, so there are some differences in Raman spectra that capture the ‘molecular fingerprint’. In addition, individual differences between patients may also lead to differences in the magnitude of the subtracted spectra. In our future work, we will expand the sample size, on one hand, to obtain more information about the patients and explore the reasons for such differences and, on the other hand, extract more features of the spectra of different tissues to further improve the accuracy and sensitivity of the classification model.

We also found that the peaks in the Raman spectra of TSCC of different histological grade groups showed some unexplained changes. The intensities of peaks at 1,001, 1,152, and 1,518 cm^−1^ increased in the spectra of MDSCC compared to PDSCC, but all these intensities of peaks decreased in the spectra compared to WDSCC. The intensities of peaks did not always increase with increasing pathologic grading. This result suggests that histological observations and pathological manifestations do not represent all biochemical changes and molecular alterations. When comparing the difference and discrimination of Raman spectra of different histologic grades of TSCC and normal muscle tissues, the subtracted spectrum showed some significant increases in the peaks at Raman shifts from 600 to 1,000 cm^−1^ in WDSCC and MDSCC groups compared to normal muscle tissue, while it showed a significant decrease in the PDSCC group compared to normal muscle tissue. When comparing the difference and discrimination of Raman spectra of PDSCC to WDSCC and MDSCC, the subtracted spectra both showed only increases in the peaks at Raman shifts from 600 to 1800 cm^−1^. However, the subtracted spectrum showed some increases and decreases when comparing WDSCC to MDSCC. These results suggest that the molecular structure and material alterations of PDSCC are more different from WDSCC and MDSCC, which may also suggest that the biological metabolism or behavior of PDSCC is significantly different. In addition, the results of the histogram of discrimination scores of TSCC of different histological grades and normal muscle show increasing differences between WDSCC, MDSCC, PDSCC, and normal muscle tissue, which may be related to the degree of differentiation of the tumor tissue. Meanwhile, the results can indirectly prove that Raman spectroscopy can be used to identify different pathological grades of TSCC.

Although we deduce that the developed PCA–LDA classification model can facilitate the objective and automated evaluation of frozen tissue sections, our conclusions are drawn from a dataset limited in size. Therefore, we aim to expand our collection of TSCC specimens of varying pathological grades in order to develop a more reliable diagnostic classification model, which will aid in the enhanced assessment of the deep surgical margins.

## 5 Conclusion

Raman spectroscopy can detect alterations in ‘molecular fingerprints’ caused by changes in biochemical composition and histopathological structures. This study demonstrates that a classification model based on differences in Raman spectroscopy can identify cancerous and muscular tissues in the region of the deep surgical margins of TSCC and that this approach shows great potential to assist in the rapid intraoperative detection and assessment of deep surgical margins of TSCC.

## Data Availability

The original contributions presented in the study are included in the article/Supplementary Material; further inquiries can be directed to the corresponding author.

## Supplementary material

SUPPLEMENTARY FIGURE S1 Raman spectra of TSCC and normal muscle for each patient.

SUPPLEMENTARY FIGURE S2 Subtracted Raman spectra of TSCC and normal muscle for each patient.
