# Supplementary figures and images for: Intraoperative rapid assessment of the deep muscle surgical margin of tongue squamous cell carcinoma via Raman spectroscopy
Source: Front Bioeng Biotechnol. 2024 Oct 8;12:1480279. doi: 10.3389/fbioe.2024.1480279 (PMC11493737; doi:10.3389/fbioe.2024.1480279)

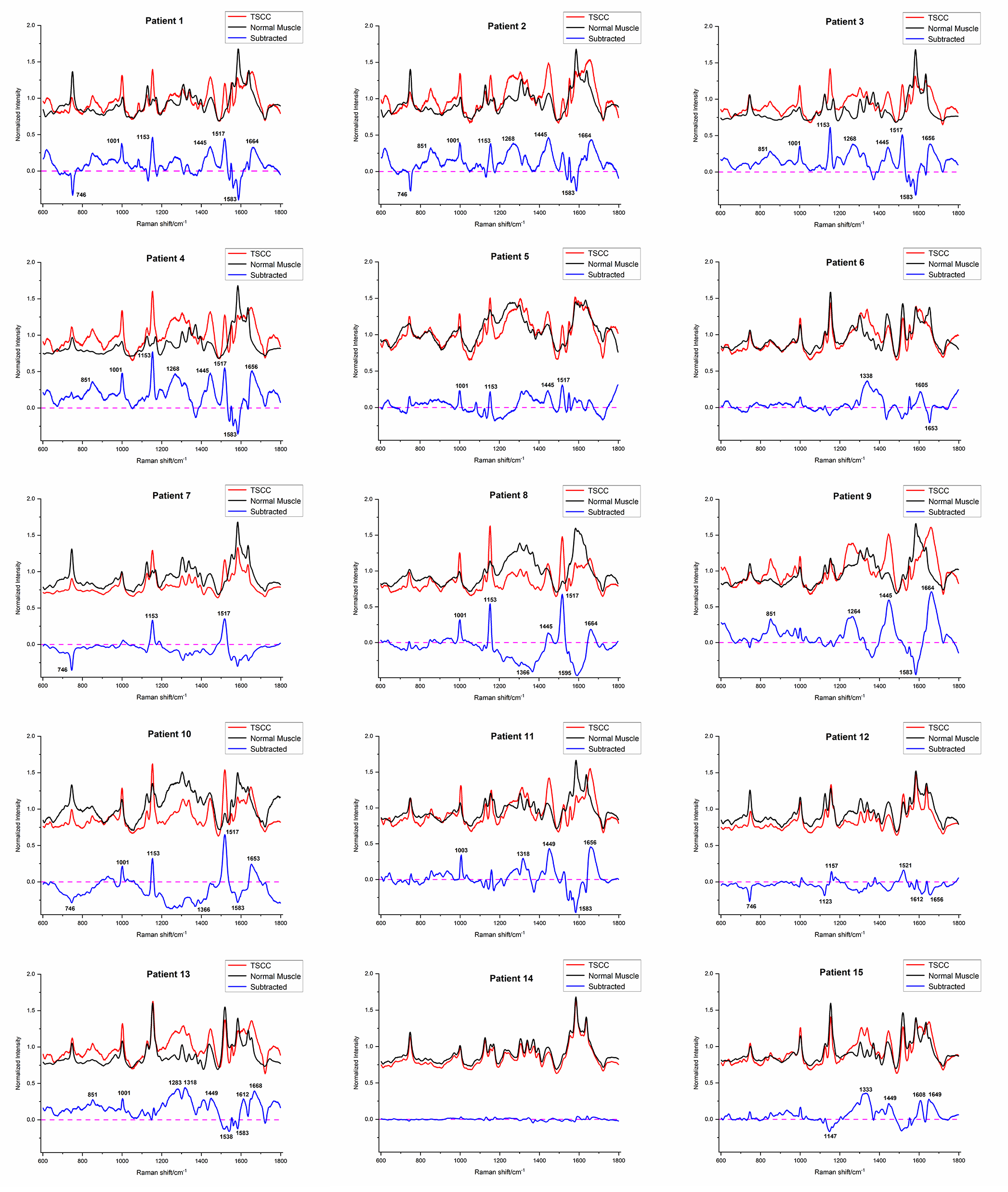

Supplement: Supplementary file 1 [file Image1.JPEG]

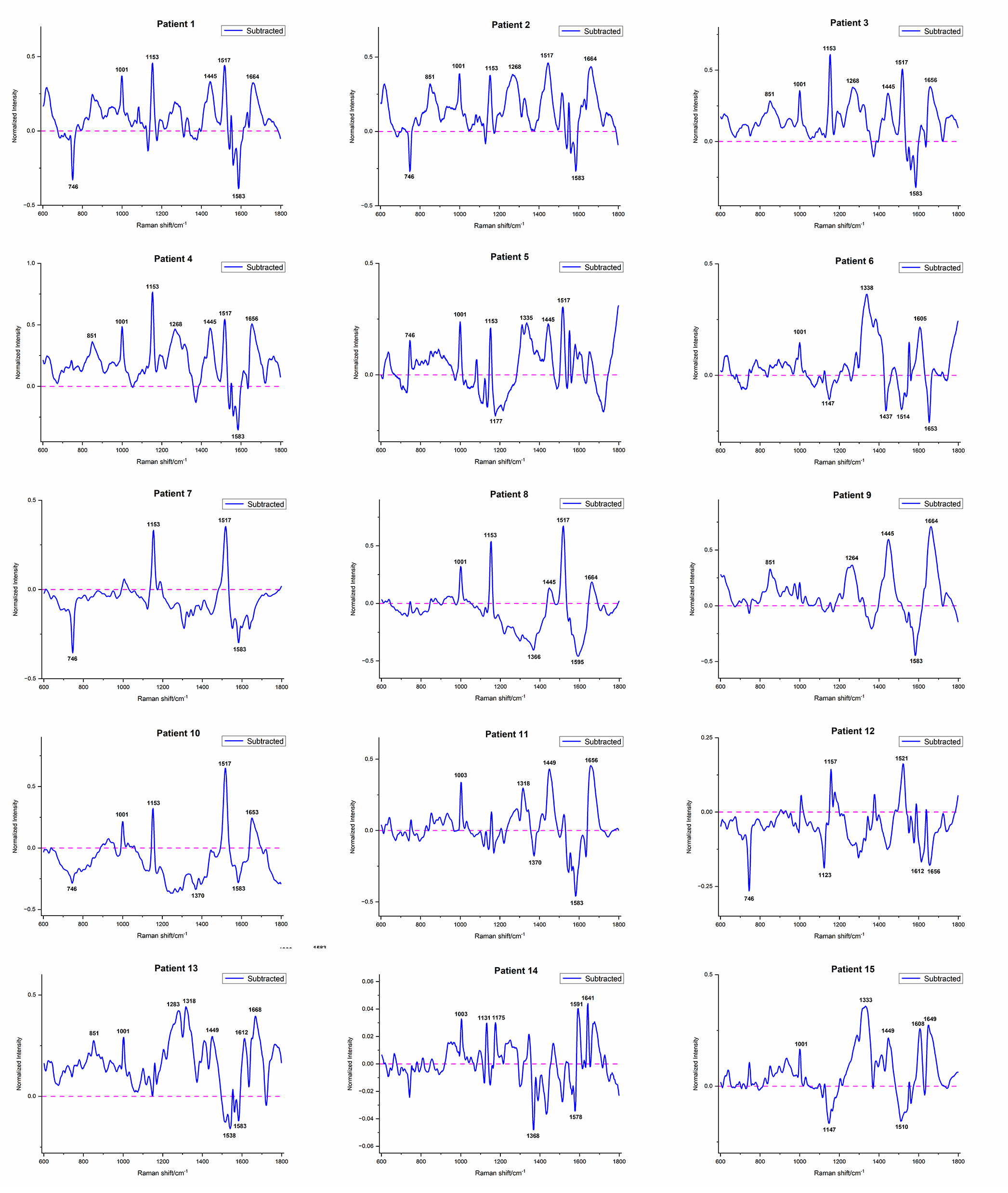

Supplement: Supplementary file 3 [file Image2.JPEG]
